# High-dose four-factor prothrombin complex concentrate for vitamin K antagonist reversal before urgent surgery: subanalysis of the LEX-209 randomized, active-controlled trial

**DOI:** 10.1016/j.rpth.2026.106879

**Published:** 2026-07-28

**Authors:** Ravi Sarode, Joshua N. Goldstein, Gregory Simonian, Doris Hinterberger, Dmitrii Matveev, Michelle Gareis, Truman J. Milling

**Affiliations:** 1 Division of Transfusion Medicine and Hemostasis, Departments of Pathology and Internal Medicine (Hematology/Oncology), the University of Texas Southwestern Medical Center, Dallas, Texas, USA; 2 Department of Emergency Medicine, Mass General Brigham, Harvard Medical School, Boston, Massachusetts, USA; 3 Divison of Vascular Surgery, Heart and Vascular Hospital, Hackensack University Medical Center, Hackensack, New Jersey, USA; 4 Octapharma Pharmazeutika Produktionsges.m.b.H, Vienna, Austria; 5 Departments of Neurology and Surgery and Perioperative Care, Dell Medical School, University of Texas at Austin, Austin, Texas, USA

**Keywords:** hemostasis, international normalized ratio, prothrombin complex concentrates, thromboembolism, warfarin

## Abstract

**Background:**

Four-factor prothrombin complex concentrates (4F-PCCs) reverse vitamin K antagonist (VKA)-induced acquired coagulation factor deficiency. However, limited data exist on the benefits and risks of 4F-PCC treatment in patients with supratherapeutic international normalized ratio (INR) values (>6), for whom a high dose of 4F-PCC (50 IU/kg) is indicated.

**Objective:**

To assess the hemostatic efficacy and safety of high-dose 4F-PCC in patients on VKA therapy before urgent surgery with an INR > 6.

**Methods:**

The LEX-209 Phase 3 randomized, active-controlled study (NCT02740335) enrolled adults taking VKAs before urgent surgery with a substantial bleeding risk. Patients were randomized to either investigational or control 4F-PCC. This post hoc subanalysis included patients with baseline INR > 6, who were given high-dose 4F-PCC (50 IU/kg). Outcomes were analyzed according to original treatment assignment. The primary endpoint was hemostatic efficacy assessed by a blinded Independent Endpoint Adjudication Board.

**Results:**

Twenty-three patients had an INR > 6 (mean ± SD INR, 9.1 ± 3.2). The mean ± SD dose of 4F-PCC administered was similar in the investigational (*n* = 15) and control (*n* = 8) 4F-PCC groups (49.7 ± 1.2 IU and 49.2 ± 2.3 IU of factor IX/kg, respectively). Effective hemostasis was achieved in 100% of patients in both groups, with adequate increments in vitamin K-dependent factors. No drug-related treatment-emergent adverse events or thromboembolic events were reported.

**Conclusions:**

In this small sample, 4F-PCC, at the maximum permitted dose, was effective for VKA reversal in patients requiring urgent surgery, with no adverse safety signal observed.

## Essentials

• Prothrombin complex concentrates (PCCs) reverse vitamin K antagonist (VKA) blood anticoagulants.
• We studied PCC treatment in people with excessive anticoagulation from VKAs who needed surgery.
• High-dose PCC restored normal blood clotting in people with excessive anticoagulation.
• No side effects were linked to PCC use, but the small study size limits safety conclusions.

## Graphical abstract

**Figure:**
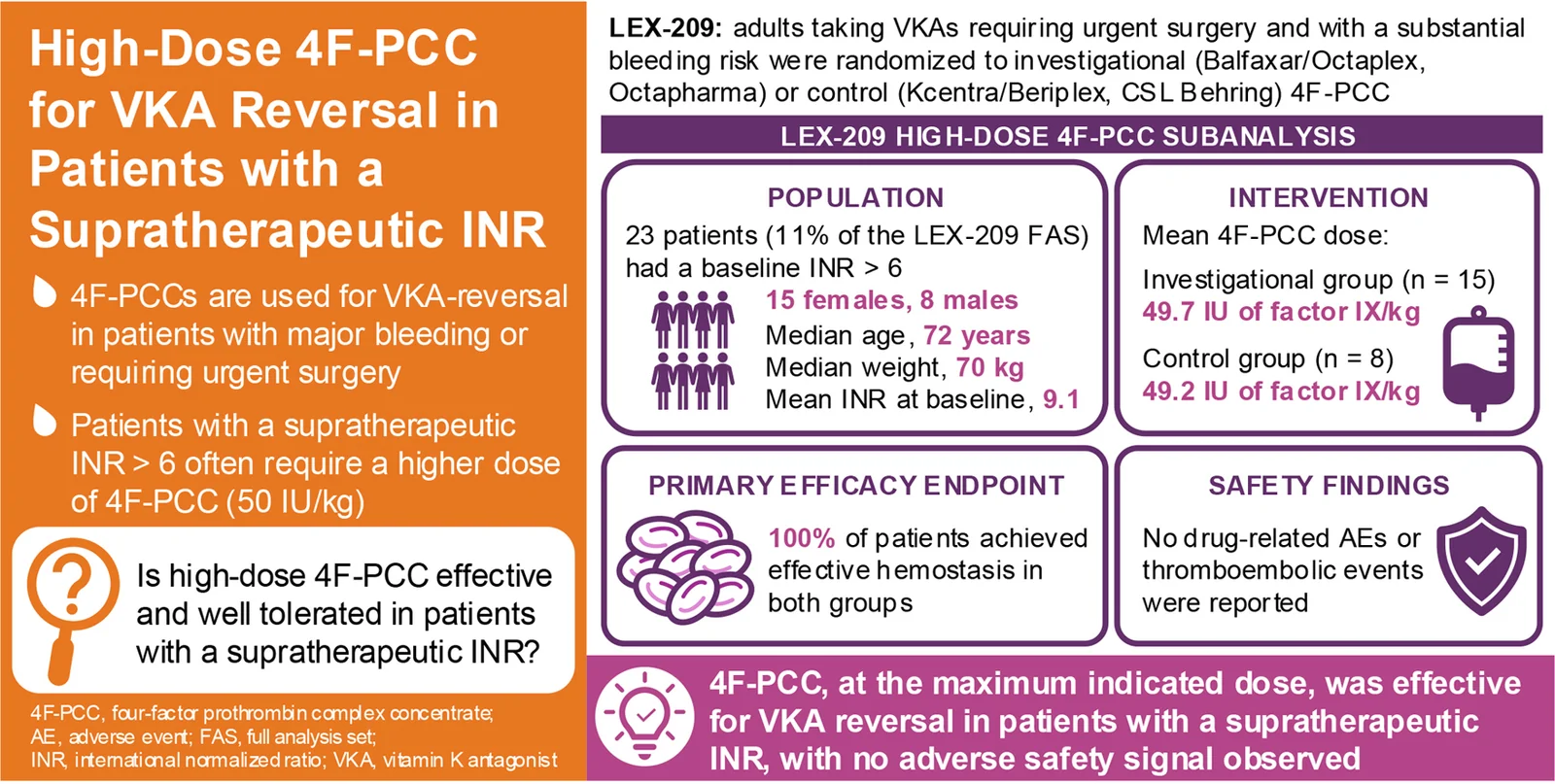

## 1 Introduction

Despite the widespread use of direct oral anticoagulants, vitamin K antagonists (VKAs) remain the preferred anticoagulants in many clinical settings, including patients with mechanical heart valves, left ventricular assist devices, or triple-positive antiphospholipid antibody syndrome [[1], [2], [3]]. Maintaining the international normalized ratio (INR) within the target range (typically 2.0–3.0) during VKA therapy is challenging due to the narrow therapeutic window and variable dose response, and overanticoagulation is frequently encountered [4,5]. For example, in a large international warfarin trial, patients spent a mean 15.7% of time with an INR above the therapeutic range [6].

VKA-associated overanticoagulation (INR > 3.0) is associated with increased morbidity and mortality, largely due to bleeding-related complications, with bleeding risk approximately doubling for each one-point increase in INR above 3.0 [4,7,8]. Accordingly, VKA-treated patients presenting with major bleeding or needing urgent surgery require rapid reversal of VKA-induced acquired coagulation factor deficiency. Four-factor prothrombin complex concentrates (4F-PCCs) are the current standard of care for rapid VKA reversal compared with plasma transfusion, owing to the targeted replacement of deficient factors and improved safety and efficacy profiles [9]. Current prescribing recommendations specify the highest 4F-PCC dose (50 IU/kg) for patients with markedly elevated INR > 6 [10], in whom factor depletion is expected to be greatest and rapid restoration of hemostatic capacity is required.

While 4F-PCCs have been evaluated for VKA reversal in broader VKA-treated populations, limited data exist on their efficacy and safety in patients with supratherapeutic INR > 6, who are often underrepresented in clinical trials. Consequently, there is a paucity of data on the balance between hemostatic efficacy and thromboembolic risk of high-dose 4F-PCC.

The LEX-209 trial was a Phase 3 study evaluating an investigational 4F-PCC (Balfaxar/Octaplex, Octapharma) compared with the US Food and Drug Administration (FDA)-approved control 4F-PCC (Kcentra/Beriplex, CSL Behring) in patients requiring rapid VKA reversal before urgent surgery with a significant bleeding risk. The trial demonstrated that the investigational 4F-PCC was noninferior to the control 4F-PCC in achieving effective hemostasis (proportion difference, 0.001; *P* < .001) [11]. However, because of the lack of data in patients with supratherapeutic INR to date, further characterization of outcomes in this subgroup requiring high-dose 4F-PCC in LEX-209 is warranted.

The objective of this subanalysis was to evaluate the hemostatic efficacy and safety of high-dose 4F-PCC in patients with INR > 6 enrolled in the LEX-209 trial, addressing an important knowledge gap.

## 2 Methods

### 2.1 Study design and patient selection

LEX-209 (ClinicalTrials.gov identifier: NCT02740335) was a prospective, randomized, active-controlled, multicenter Phase 3 trial conducted at 24 hospitals in Belarus, Georgia, Romania, Russia, Ukraine, and the United States. The trial enrolled patients aged ≥18 years receiving VKA therapy who required urgent surgery and were at risk of significant perioperative blood loss of at least 50 mL during the procedure. Institutional review boards or ethics committees approved the study protocol at each participating site, and all participants provided written informed consent [11]. This exploratory post hoc subgroup analysis included all trial participants who had a baseline INR > 6. No new randomization was performed for this analysis; instead, all patients with baseline INR > 6 were identified from the original trial after completion.

### 2.2 Intervention and dosing

In LEX-209, each patient received a single intravenous infusion of either investigational 4F-PCC (Balfaxar/Octaplex, Octapharma) or control 4F-PCC (Kcentra/Beriplex, CSL Behring) immediately before surgery [11]. All patients in the INR > 6 subgroup were given the maximum protocol-specified dose of 50 IU (of factor IX units) per kg of body weight, with body weight capped at 100 kg, corresponding to a maximum total 4F-PCC dose of 5000 IU. Vitamin K was administered concomitantly, as per local institutional protocols [11].

### 2.3 Outcomes

The primary efficacy outcome as defined in LEX-209 was hemostatic efficacy at the end of surgery, evaluated on a four-point ordinal scale (excellent, good, moderate, or none) by a blinded Independent Endpoint Adjudication Board. For analysis purposes, “effective” hemostasis was defined as a rating of excellent or good, whereas moderate or none was considered “ineffective.” Secondary efficacy endpoints included the proportion of patients with an INR ≤ 1.5 at 30 (±15) minutes post infusion**,** serial changes in vitamin K-dependent factor levels, and the proportion of patients receiving intraoperative red blood cells [11]. Identical outcome definitions and assessment criteria from the full LEX-209 trial were applied to this subgroup analysis. Safety assessments monitored treatment-emergent adverse events (TEAEs), including thromboembolic events (TEEs), with investigators assessing the relationship of TEAEs to 4F-PCC. Safety data were continuously monitored throughout the study by an Independent Data Monitoring Committee [11]. All efficacy and safety data from the subgroup were extracted from the original trial records; no additional interventions or follow-up beyond the parent study were undertaken for this post hoc analysis.

### 2.4 Statistical analysis

Statistical analyses were primarily descriptive and exploratory. Continuous variables were summarized using means and standard deviations or medians and ranges, as appropriate. Categorical outcomes were described as counts and percentages for each treatment group. Differences in efficacy proportions between the investigational and control 4F-PCC subgroups were estimated with two-sided 95% confidence intervals, calculated by the Farrington–Manning test. No formal hypothesis testing was planned for this subgroup analysis, which was not prespecified or powered for statistical comparisons. Statistical analyses were performed using SAS (version 9.3 or higher; SAS Institute Inc), consistent with the approach for the original trial analysis [11].

## 3 Results and Discussion

Of the 208 randomized patients in LEX-209, 23 (11%) had a baseline INR > 6 and were included in this subgroup analysis. Of these, 15 patients received the investigational 4F-PCC and 8 received the control 4F-PCC. Overall, the subgroup included 15 females and 8 males, all White and non-Hispanic or Latino, with a mean baseline INR of 9.1 (SD ± 3.2; range, 6.2-21.1), indicating an overanticoagulated group. The median age was 72 years (range, 44-90 years), and the median body weight was 70 kg (range, 50-115 kg). The location of surgery, the 4F-PCC dose administered, and the time from the initiation of the 4F-PCC infusion to the start of the surgery for these patients are presented in the Table.

**Table:** Surgery and treatment characteristics, and efficacy and safety results.

Parameter	Investigational group (*n* = 15)	Control group (*n* = 8)
Surgery and treatment characteristics		
Location of surgery, *n* (%)		
Gastrointestinal	8 (53.3)	5 (62.5)
Musculoskeletal	3 (20.0)	0 (0)
Cardiovascular	0 (0)	1 (12.5)
Other	4 (26.7)	2 (25.0)
4F-PCC dose, IU factor IX/kg		
Mean ± SD	49.7 ± 1.2	49.2 ± 2.3
Range	45.5–50.0	43.5–50.0
Time from 4F-PCC infusion start to surgery start (min)		
Mean ± SD	95.3 ± 48.8	83.8 ± 29.7
Efficacy results		
Patients with hemostatic efficacy, as assessed by the IEAB, *n* (%)	15 (100)	8 (100)
Patients with an INR ≤ 1.5 at 30 min after the end of infusion, *n* (%)	14 (93.3)^a^	8 (100)
Proportion difference	−0.067	
95% CI for difference	–0.247, 0.114	
Safety results		
TEAEs^b^		
*n*	35	10
Patients, *n* (%)	14 (93.3)	7 (87.5)
TEAEs related to study drug, *n*	0	0
TEEs related to study drug, *n*	0	0

4F-PCC, four-factor prothrombin complex concentrate; CI, confidence interval; IEAB, Independent Endpoint Adjudication Board; INR, international normalized ratio; TEAE, treatment-emergent adverse event; TEE, thromboembolic event.

a The remaining patient had an INR of 1.52 at 30 min after the end of infusion.

b All TEAEs were assessed by the investigator as “not related” to study drug, except for one event of “pain in extremity” in the investigational group which was assessed as “unlikely” to be related to study drug.

There was 100% hemostatic efficacy, as assessed by the Independent Endpoint Adjudication Board, in patients with a baseline INR > 6. At 30 minutes after the end of infusion, all patients had an INR ≤ 1.5, except for one patient in the investigational arm, who had an INR of 1.52 (Table). This patient underwent surgery without excessive bleeding. All patients achieved adequate increments in vitamin K-dependent factor levels within 30 minutes post infusion (Figure). One patient in the investigational group received red blood cells during surgery; no patients in either treatment group received intraoperative plasma or platelet transfusion.

**Figure:**
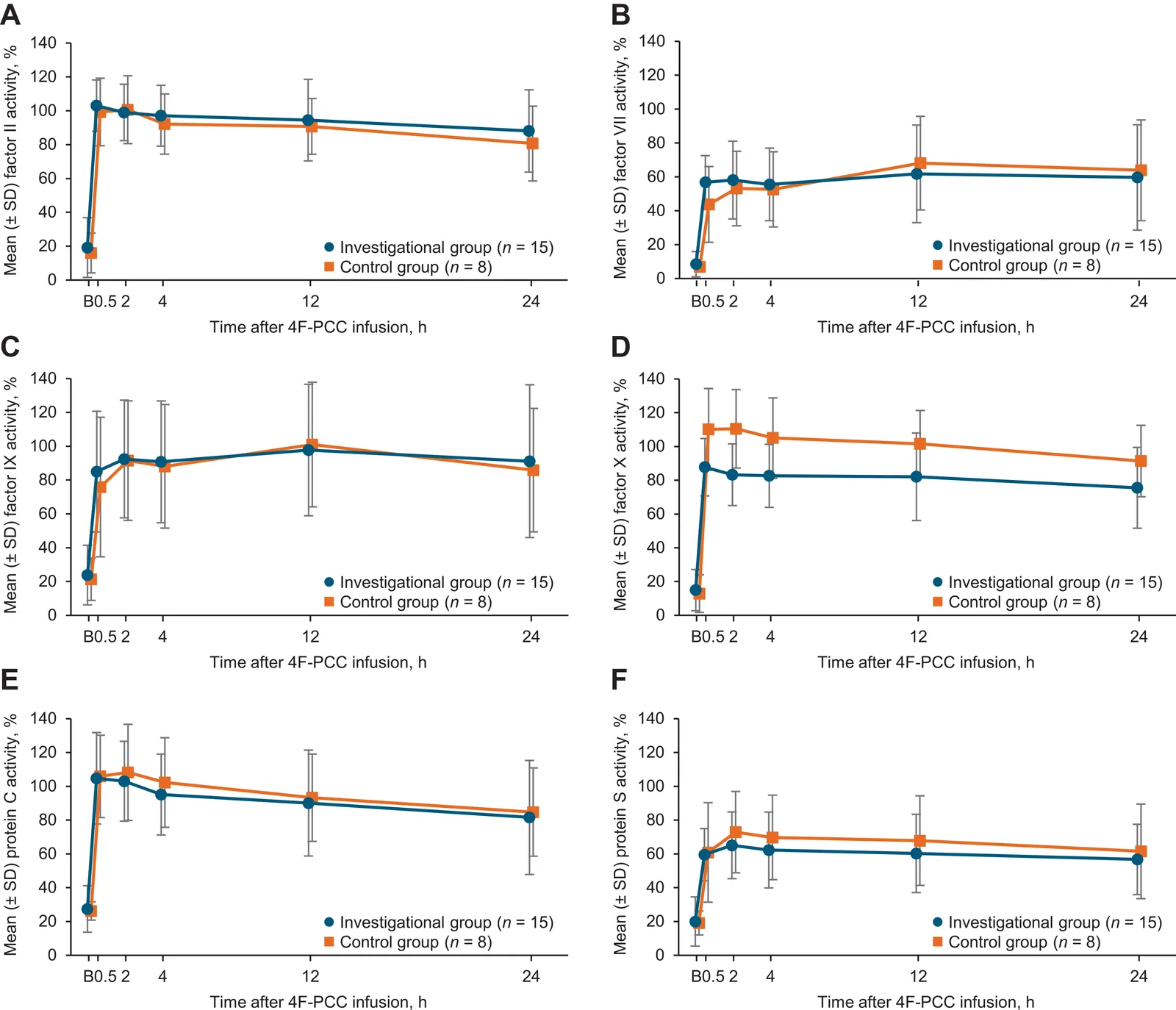
Activities of vitamin K-dependent factors over time after infusion of 4F-PCC. (A) Factor II. (B) Factor VII. (C) Factor IX. (D) Factor X. (E) Protein C. (F) Protein S. To make them easier to distinguish, data point markers and error bars (which represent SDs) for the investigational group and the control group are offset at each time point on the horizontal axis. Two patients in the investigational group and one patient in the control group were missing data for factor X activity at baseline. One patient in the control group was missing data for factor II and factor X activity at 0.5 hour after infusion. 4F-PCC, four-factor prothrombin complex concentrate; B, baseline.

Similar proportions of patients in the investigational and control groups experienced TEAEs, none of which were deemed related to the study drug (Table). In the investigational group, the most common TEAEs were procedural pain (*n* = 9 [60.0%]) and anemia (*n* = 3 [20.0%]). In the control group, the most common TEAEs were procedural pain (*n* = 4 [50.0%]) and postoperative wound complications (*n* = 3 [37.5%]). There were three TEEs in two patients in the investigational group. First, an 89-year-old patient (baseline INR = 21.1) who underwent small intestinal resection had cerebral infarction at 12 days after receiving 4F-PCC and pulmonary embolism at 37 days post infusion, resulting in death on the same day. Oral anticoagulation was not restarted postoperatively. Relevant medical history included hypertension, congestive heart failure, ischemic heart disease, permanent atrial fibrillation, and nonrheumatic mitral valve stenosis and insufficiency with secondary pulmonary hypertension. Second, a 90-year-old patient (baseline INR = 6.8) who underwent osteosynthesis developed myocardial ischemia and died at 30 days after receiving 4F-PCC. Postoperatively, the patient had received an oral anticoagulant prescription. Relevant medical history included ischemic heart disease, arterial hypertension, chronic cardiac failure, and atrial fibrillation. These TEEs were deemed unrelated to the study drug by the investigators and the Independent Data Monitoring Committee.

This subgroup analysis of the LEX-209 Phase 3 trial assessed the efficacy and safety of high-dose 4F-PCC for rapid VKA reversal in patients presenting with baseline INR > 6 before urgent surgery. Both the investigational and control 4F-PCCs were found to be highly effective, with all patients achieving hemostatic efficacy, and no adverse safety signals were observed.

These findings are clinically significant because patients with supratherapeutic INR values represent a high-risk subgroup. This is due to the high baseline anticoagulation burden in these patients, characterized by severe reductions in key factors II and X and the requirement for maximal 4F-PCC dosing (50 IU/kg). Consequently, timely and reliable VKA reversal is essential to reduce the risk of perioperative bleeding and associated complications. Prior studies evaluating 4F-PCC efficacy have not included subanalyses of patients with supratherapeutic INR [12,13], and thus, data to guide management in this cohort have remained limited. The observation that INR correction to ≤1.5 at 30 minutes after the end of infusion was achieved in nearly all patients, associated with adequate increments of vitamin K-dependent factor levels, further supports the rapid onset of action and consistent pharmacodynamic profiles of both treatments.

Often, there is concern about TEEs with 4F-PCC administration in patients with an underlying hypercoagulable state, especially at the maximum dose. In this subanalysis, three TEEs occurred in two patients in the investigational group, all of which were deemed unrelated to the study drug. One patient (baseline INR = 21.1), for whom oral anticoagulation was not restarted postoperatively, experienced two TEEs at 12 and 37 days after 4F-PCC infusion; the other patient (baseline INR = 6.8) experienced a TEE at 30 days post infusion and, although a prescription was issued, reinitiation of oral anticoagulant therapy postoperatively was not confirmed. The half-lives of infused vitamin K-dependent factors are up to 72 hours [11], and real-world data suggest that the majority (∼60%) of TEEs after 4F-PCC administration occur within the first 7 days after treatment [14]. Additionally, most TEEs occur in patients who have not resumed anticoagulation therapy, often despite having underlying thromboembolic risk factors. For example, in post hoc analyses of two randomized trials, only 21% of 4F-PCC-treated patients who experienced TEEs had restarted VKA therapy [15]. Thus, late-occurring TEEs (≥12 days post infusion) after 4F-PCC treatment likely reflect underlying patient-specific risk factors rather than a direct drug effect. Here, both patients were of advanced age with multiple cardiovascular comorbidities and oral anticoagulation was either not restarted or not confirmed as restarted, increasing the risk of TEEs. All three TEEs in this subanalysis were reported in the investigational group; however, given the small, imbalanced sample and the exploratory nature of this analysis, any trends in TEE incidence should be interpreted with caution. Rather, while no causal link with 4F-PCC was established for these events, their occurrence underscores the need for further data.

This analysis had several strengths. The use of centralized, blinded adjudication for the primary outcome minimized assessment bias. Additionally, the dosing regimen was standardized and protocol-driven, and the patient population reflected real-world clinical scenarios involving urgent procedural intervention. However, some limitations must be acknowledged. This analysis was not prespecified, and the study was not powered by design to formally evaluate outcomes in this subgroup of patients. Accordingly, any observed differences or lack thereof in efficacy or adverse events are exploratory and descriptive only. The limited sample size and imbalance between the treatment groups further preclude any definitive conclusions from being drawn. Nevertheless, the observed consistency of findings across both treatment groups may provide some reassurance for clinicians considering high-dose 4F-PCC in patients with supratherapeutic INRs until further prospective evidence becomes available.

## 4 Conclusions

In conclusion, both the investigational and control 4F-PCCs demonstrated hemostatic efficacy in patients with supratherapeutic INR > 6 and significant bleeding risk undergoing urgent surgery. No adverse safety signal was identified in this subanalysis; however, given the small sample, rare events like TEEs cannot be excluded, and the findings underscore the potential importance of timely resumption of anticoagulation where clinically appropriate. Overall, these results support the use of high-dose 4F-PCC for the rapid reversal of VKA in overanticoagulated patients, although further study of this high-risk subgroup is warranted.

## References

[1] Eikelboom J.W., Connolly S.J., Brueckmann M., Granger C.B., Kappetein A.P., Mack M.J. (2013). Dabigatran versus warfarin in patients with mechanical heart valves. N Engl J Med.

[2] Andreas M., Moayedifar R., Wieselthaler G., Wolzt M., Riebandt J., Haberl T. (2017). Increased thromboembolic events with dabigatran compared with vitamin K antagonism in left ventricular assist device patients: a randomized controlled pilot trial. Circ Heart Fail.

[3] Rodziewicz M., D'Cruz D.P. (2020). An update on the management of antiphospholipid syndrome. Ther Adv Musculoskelet Dis.

[4] Dentali F., Crowther M.A. (2008). Management of excessive anticoagulant effect due to vitamin K antagonists. Hematol Am Soc Hematol Educ Program.

[5] Gallus A.S., Baker R.I., Chong B.H., Ockelford P.A., Street A.M. (2000). Consensus guidelines for warfarin therapy. Recommendations from the Australasian Society of Thrombosis and Haemostasis. Med J Aust.

[6] Singer D.E., Hellkamp A.S., Piccini J.P., Mahaffey K.W., Lokhnygina Y., Pan G. (2013). Impact of global geographic region on time in therapeutic range on warfarin anticoagulant therapy: data from the ROCKET AF clinical trial. J Am Heart Assoc.

[7] Shoeb M., Fang M.C. (2013). Assessing bleeding risk in patients taking anticoagulants. J Thromb Thrombolysis.

[8] Marie I., Leprince P., Menard J.-F., Tharasse C., Levesque H. (2012). Risk factors of vitamin K antagonist overcoagulation. QJM.

[9] Karkouti K., Gareis M., Li C., Brandstätter H., Pichotta A., Shah T.M. (2025). Twenty years of the four-factor prothrombin complex concentrate Octaplex/Balfaxar: a narrative review. Transfus Apher Sci.

[10] Octapharma (2023). BALFAXAR: Highlights of prescribing information. https://www.fda.gov/files/vaccines%2C%20blood%20%26%20biologics/published/Package-Insert-BALFAXAR.pdf.

[11] Sarode R., Goldstein J.N., Simonian G., Hinterberger D., Matveev D., Gareis M. (2024). Vitamin K antagonist reversal for urgent surgery using 4-factor prothrombin complex concentrates: a randomized clinical trial. JAMA Netw Open.

[12] Sarode R., Milling T.J., Refaai M.A., Mangione A., Schneider A., Durn B.L. (2013). Efficacy and safety of a 4-factor prothrombin complex concentrate in patients on vitamin K antagonists presenting with major bleeding: a randomized, plasma-controlled, phase IIIb study. Circulation.

[13] Goldstein J.N., Refaai M.A., Milling T.J., Lewis B., Goldberg-Alberts R., Hug B.A. (2015). Four-factor prothrombin complex concentrate versus plasma for rapid vitamin K antagonist reversal in patients needing urgent surgical or invasive interventions: a phase 3b, open-label, non-inferiority, randomised trial. Lancet.

[14] Makhoul T., Kelly G., Kersten B., Nadler M., Zammit C.G., Jones C.M.C. (2020). Incidence of thromboembolic events following administration of four-factor prothrombin complex concentrate (4F-PCC) for oral anticoagulation reversal. Thromb Res.

[15] Milling T.J., Refaai M.A., Goldstein J.N., Schneider A., Omert L., Harman A. (2016). Thromboembolic events after vitamin K antagonist reversal with 4-factor prothrombin complex concentrate: exploratory analyses of two randomized, plasma-controlled studies. Ann Emerg Med.

