# Supplementary material for: High-dose four-factor prothrombin complex concentrate for vitamin K antagonist reversal before urgent surgery: subanalysis of the LEX-209 randomized, active-controlled trial
Source: Res Pract Thromb Haemost. 2026 Jul 28;10(5):106879. doi: 10.1016/j.rpth.2026.106879 (PMC13507540; doi:10.1016/j.rpth.2026.106879)
Supplement: Supplementary file 1 [file mmc1.pdf]

High-Dose 4F-PCC for VKA Reversal in Patients with a Supratherapeutic INR

- 4F-PCCs are used for VKA-reversal in patients with major bleeding or requiring urgent surgery
- Patients with a supratherapeutic INR > 6 often require a higher dose of 4F-PCC (50 IU/kg)

Is high-dose 4F-PCC effective and well tolerated in patients with a supratherapeutic INR?

4F-PCC, four-factor prothrombin complex concentrate;
AE, adverse event; FAS, sull analysis set;
INR, international normalized ratio; VKA, vitamin K antagonist

LEX-209: adults taking VKAs requiring urgent surgery and with a substantial bleeding risk were randomized to investigational (Balfaxar/Octaplex, Octapharma) or control (Kcentra/Beriplex, CSL Behring) 4F-PCC

LEX-209 HIGH-DOSE 4F-PCC SUB-ANALYSIS

POPULATION

23 patients (11% of the LEX-209 FAS) had a baseline INR > 6

15 females, 8 males
Median age, **72 years**
Median weight, **70 kg**
Mean INR at baseline, **9.1**

INTERVENTION

Mean 4F-PCC dose:

Investigational group (n = 15)
49.7 IU of factor IX/kg

Control group (n = 8)
49.2 IU of factor IX/kg

PRIMARY EFFICACY ENDPOINT

100% of patients achieved effective hemostasis in both groups

SAFETY FINDINGS

No drug-related AEs or thromboembolic events were reported

4F-PCC, at the maximum indicated dose, was effective for VKA reversal in patients with a supratherapeutic INR, with no adverse safety signal observed
